# Lipid status association with 25-hydroxy vitamin D: Cross sectional study of end stage renal disease patients

**DOI:** 10.2478/jomb-2019-0032

**Published:** 2020-09-02

**Authors:** Neda Milinković, Marija Sarić, Snežana Jovičić, Duško Mirković, Višnja Ležaić, Svetlana Ignjatović

**Affiliations:** 1 University of Belgrade, Faculty of Pharmacy, Department of Medical Biochemistry, Belgrade; 2 University of Belgrade, Faculty of Medicine, Department of Nephrology

**Keywords:** lipid parameters, 25-hydroxy vitamin D, end stage renal disease, dialysis, lipidni parametri, 25-hidroksi vitamin D, krajnji stadijum bolesti bubrega, dijaliza

## Abstract

**Background:**

Some observational studies indicate an association of 25-hydroxy vitamin D (25(OH)D) insufficiency and atherogenic cholesterol concentrations. The aim of this study was to investigate relationship between 25(OH)D concentrations and lipid parameters in end stage renal disease (ESRD) patients, separately for predialysis, hemodialysis and peritoneal dialysis patients.

**Methods:**

We have adjusted 25(OH)D concentrations for seasonal variability with cosinor analysis, and performed all further analysis using these corrected 25(OH)D concentrations. Concentrations of 25(OH)D and the lipid parameters were determined in 214 ESRD patients and 50 control group participants. The analysis included the measurement of 25(OH)D by HPLC, apolipoprotein (Apo) AI, ApoB and Lp(a) by nephelometry, total cholesterol (TC), high-density lipoprotein cholesterol (HDL-C), low-density lipoprotein cholesterol (LDL-C) and triglyceride (TG) by spectrophotometry and manually calculated ApoB/ApoAI and LDL-C/HDL-C ratio.

**Results:**

ESRD patients with adjusted 25(OH)D concentrations of 50 nmol/L had significantly higher TC (P = 0.005) and ApoAI (P = 0.049). Significantly higher HDLC (P = 0.011) and ApoAI (P = 0.020) were found in hemodialysis patients with the 25(OH)D concentrations of 50 nmol/L. The other analyzed lipid parameters differed significantly between predialysis, hemodialysis and peritoneal dialysis patients with 25(OH)D concentrations of < 50 nmol/L.

**Conclusions:**

Our study indicate the significant relationship between 25(OH)D repletion and optimal concentrations of lipid parameters in ESRD patients. Further research is necessary to explain whether joint evaluation of vitamin D status and lipid abnormalities could improve cardiovascular outcome in ESRD patients.

## Abstract (sr)

**Uvod:**

Opservacione studije ukazuju na povezanost nedostatka 25-hidroksi vitamin D (25(OH)D) i koncentracija aterogenog holesterola. Cilj ove studije bio je da se ispita međusobna povezanost 25(OH)D i lipidnih parametara kod bolesnika sa krajnjim stadijumom bolesti bubrega (ESRD) posebno kod predijaliznih, bolesnika na hemodijalizi i peritonalnoj dijalizi.

**Metode:**

Podesili smo koncentracije 25(OH)D za sezonsku varijaciju koristeći kosinor analizu, i u daljem ispitivanju smo koristili korigovane koncentracije 25(OH)D. Koncentracije 25(OH)D i lipidnih parametara su određene za 214 ESRD bolesnika i 50 učesnika kontrolne grupe. Analizirali smo koncentracije 25(OH)D metodom HPLC, apolipoproteina (Apo) AI, ApoB i Lp(a) nefelometrijski, ukupnog holesterola (TC), lipoproteina velike gustine (HDL-C), lipoproteina male gustine (LDL-C) i triglicerida (TG) spektro fotometrijski i ručno preračunavali indekse ApoB/ApoAI i LDL-C/HDLC.

**Rezultati:**

ESRD bolesnici sa korigovanim 25(OH)D 50 nmol/L su imali značajno viši TC (P = 0,005) i ApoAI (P = 0,049). Značajno više koncentracije HDL-C (P = 0,011) i ApoAI (P = 0,020) smo dobili kod hemodijaliznih bolesnika sa 25(OH)D koncentracijama 50 nmol/L. Ostali analizirani lipidni parametri su bili značajno različiti između analiziranih grupa bolesnika sa 25(OH)D koncentracijama < 50 nmol/L.

**Zaključak:**

Naša studija ukazuje na značajnu povezanost dovoljnih nivoa 25(OH)D i optimalnih koncentracija lipidnih parametara kod ESRD bolesnika. Potrebna su dalja istraživanja da bi se objasnilo da li zajednička procena statusa vitamina D i lipidnih abnormalnosti mogu da poboljšaju kardiovaskularni ishod kod ESRD bolesnika.

## Introduction

End stage renal disease (ESRD) is associated with apolipoprotein abnormalities and dyslipidemia [1] [2] [3]. Altered lipid metabolism is usually deemed to be a consequence of renal disease, but a large body of clinical and experimental studies support the fact that there is inter-dependence of vitamin D levels with serum lipid profiles in ESRD patients [4]. Some investigations have proposed that there is potential association of vitamin D binding protein (DBP) with lipoproteins in chronic renal failure, but also that significant urinary DBP losses contributing to vitamin D deficiency [5].

Literature data suggests that high 25-hydroxy vitamin D (25(OH)D) concentrations have been strongly correlated with an optimal concentrations of lipid parameters, but only a few studies take into account ESRD patients and dialysis type effects on vitamin D relationship with lipids [5] [6]. In support of this, in vitro studies have proved that 25(OH)D inhibits 3-hydroxy-3-methyl-glutaryl-coenzyme A reductase (HMGCoA reductase), the major cholesterol synthesis enzyme and therefore reduces total cholesterol level [7]. Vitamin D deficiency is an increasingly common diagnosis in ESRD patients and low concentrations of 25(OH)D are assocciated with many diseases, including atherogenic cholesterol concentrations. Major problem for adequate assessment of vitamin D level and its relationship with lipids lies in the fact that seasonal variation may reduce the validity of 25(OH)D as a biomarker of overall vitamin D status. It is therefore important to implement a seasonal adjustment to correct measured 25(OH)D concentrations.

Recommendation of updated clinical practice guideline for lipid management in chronic kidney disease (KDIGO - Kidney Disease Improving Global Outcomes) is to evaluate lipid status by measuring total cholesterol (TC), high-density lipoprotein cholesterol (HDL-C), low-density lipoprotein cholesterol (LDL-C) and triglycerides (TG) [8]. The most important abnormalities are an increase in serum LDL-C and TG, and a decrease in HDL-C concentrations, while TC concentrations are mostly in the normal limits. On the other hand, renal dyslipidemia is cha racterized to a greater extent by abnormal apo lipoprotein rather than recommended lipid parameters [9]. Moreover, ESRD patients usually have decreased concentrations of apolipoprotein A (ApoA) containing lipoproteins (apoAI) and increased concentrations of apolipoprotein B (ApoB) and lipo protein(a) (Lp(a)) [10] [11]. These changes consequently could lead to cardiovascular complications. Recommended therapy with statins could improve lipid and apolipoprotein status in ESRD patients, but more recent studies have explained the benefit of vitamin D therapy on this issue in relation to cardiovascular disease (CVD) [2] [12] [13].

Best screening for vitamin D status in ESRD patients is measurement of 25(OH)D [12]. On the basis of recent recommendations, a 25(OH)D concentration ranging from 50 to 75 nmol/L was defined as vitamin D repletion, a 25(OH)D concentration of < 50 nmol/L as vitamin D insufficiency and a 25(OH)D concentration of < 25 nmol/L as severe vitamin D deficiency [14]. Because ESRD patients often take vitamin D supplements for therapeutic purpose and consequently rarely exhibit severe vitamin D deficiency, in this study we have chosen concentration of 50 nmol/L for 25(OH)D as debatable. Nevertheless, a recent publication has pointed out that there is no agreement on the reference ranges for serum total 25(OH)D, which represents a problem in patient categorization [15].

The aim of this study was to evaluate the concentrations of TC, HDL-C, LDL-C, TG, ApoAI, ApoB and Lp(a) in ESRD patients in relation to control group (CG) participants and in relation to 25(OH)D concentrations (< 50 nmol/L and ≥ 50 nmol/L). In addition, we have examined the analyzed lipid parameters separately in predialysis patients (PD), chronic hemodialysis patients (HD) and in patients on continuous ambulatory peritoneal dialysis (CAPD). We intended to investigate if there were more expressive changes in the ApoB/ApoAI and LDL-C/HDL-C ratio in respect of individual lipid parameters, as well as 25(OH)D concentration.

## Materials and Methods

### Study design and sample collection

This study was conducted between February and July 2013, on PD (N = 33), HD (N = 89) and CAPD (N = 92) who were admitted and have been treated at the Clinic of Nephrology and Urology, Clinical Center of Serbia. All patients gave informed consent to participate in the study, which was approved by the Ethics Committee of the University Clinical Center, Belgrade. The CG participants (N = 50) were recruited among healthy Serbian volunteers. Selection was based on the results of laboratory tests (within the reference intervals used in the Department of Polyclinic Laboratory Diagnostics, the Center for Medical Biochemistry, the Clinical Center of Serbia), and the completed questionnaire before phlebotomy. Based on the questionnaire, we selected people with the absence of chronic or acute illness and without the use of pharmacologically active substances.

Median age for PD, HD and CAPD group of patients was 49 years (range: 24-76), 52 years (range: 26-74) and 54 years (range: 32-79), respectively. Median age for the CG was 50 years (range: 25-70). According to the KDIGO guideline and glomerular filtration rate (GFR) all the analyzed patients were categorized as G5 (Kidney failure) [16]. All PD had a creatinine clearance < 15 mL × min^−1^ × (1.73 m^2^)^−1^ and required maintenance dialysis within three months after entering the study. All HD underwent chronic haemodialysis treatment three times a week and the median time since the beginning of haemodialysis was 72 months. Median duration of CAPD was 22 months. Of all the studied patients, 9 PD, 43 HD and 25 CAPD had received oral 1 -OH D3 (0.25 μg/day). Exclusion criteria for this study were: diabetes mellitus, known or suspected liver disease and immunosuppressive therapy. Keeping in mind the seasonal variability of vitamin D concentrations, we adjusted the measured levels of 25(OH)D for seasonal differences.

### Methods

Blood samples were drawn via antecubital venipuncture after an overnight fast, in PD patients when they were on routine laboratory examinations and in HD patients blood was drawn prior to dialysis. CAPD patients continued their dialysis during sampling.

Serum 25(OH)D concentrations were determined by chromatography, using reversed phase HPLC technique with UV detection on 265 nm (ChromLineR Clinical HPLC software Version 4.20B, Chromsystems Instruments & Chemicals GmbH, Gräfelfing, Germany). We used commercial quality control samples to perform precision testing. Total precision for internal quality control samples with avarage 25(OH)D concentrations of 72.4 nmol/L and 222 nmol/L were 4.7% and 4.0%, respectively. The cosinor model was applied to baseline data, and the corrected values were used for all further analysis. Since vitamin D concentrations follow a sinusoidal seasonal pattern, we fitted a cosinor model to baseline monthly 25(OH)D concentrations. Then, we estimated each patient's adjusted 25(OH)D concentration. Period of measurement was 6 months (from February to July). To adjust seasonal 25(OH)D variation according to each patients' characteristics, we included covariates in the model (gender, age).

Sine curve is defined with an amplitude and a phase. Amplitude (A) is defined as the maximum distance of the sine wave from the mean. One can estimate the seasonal variation based on the amplitude. The phase is defined as the position of the peak on the x axis,which is equivalent to the month of the highest 25(OH)D concentration [17].

Concentrations of ApoAI, ApoB and Lp(a) were measured by an immunonephelometric method on a Behring Nephelometer II analyzer (Siemens Healthcare GmbH, Erlangen, Germany). Total cholesterol, HDL-C, LDL-C and TG were determined using spectrophotometric commercial assays on Olympus AU2700 analyser (Beckman Coulter Diagnostics, Hamburg, Germany). LDL-C concentrations were calculated by the Friedewald equation, except when triglycerides were > 4.45 mmol/L, when we used direct spectrophotometric assay.

We used a set of commercial quality control samples for internal quality control: 25-OH-Vitamin D3/D2 Serum Control Level I and Level II for 25(OH)D (Chromsystems Instruments & Chemicals GmbH, Gräfelfing, Germany), Apolipoprotein Control Serum CHD (human) and N Lp(a) Control SY for ApoAI, ApoB and Lp(a) (Siemens Healthcare GmbH, Erlangen, Germany) and OLY 1 and OLY 2 Control Serum for TC, HDL-C, LDL-C and TG (Beckman Coulter Diagnostics, Hamburg, Germany). Intra-assay CVs for all measured parameters were < 5%.

### Statistical analysis

We used one-sample Kolmogorov-Smirnov and Shapiro-Wilk test to analyze normality of distributions of the analyzed parameters. Descriptive measures are expressed as median and interquartile range (IQR). Kruskall-Walis independant samples test was applied to compare the concentration of the analyzed apolipoproteins and lipids between patients and CG, to compare the apolipoproteins and lipids concentration regarding the 25(OH)D status and to detect differences in apolipoprotein and lipid concentration between PD, HD and CAPD patients. A value of P < 0.05 was considered significant. For cosinor analysis we used R statistical softwer (version R 3.5.1) [18]. Statistical analyses were performed with SPSS version 21.0 (IBM, Chicago, USA).

## Results

**Figure 1:**
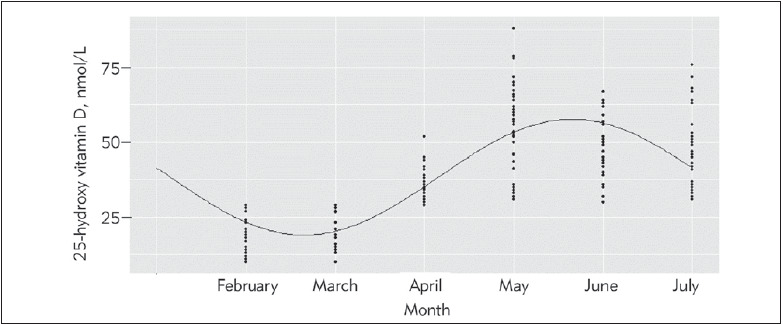
Seasonality of 25(OH)D concentrations in 214 end stage renal disease patients at base line. The overlaid curve is from an unadjusted cosinor model and shows the annual mean 25(OH)D concentration according to month of the year

Figure 1 represents the measured 25(OH)D concentrations at baseline according to the month of blood draw. Sinusoidal model closely approximated measured 25(OH)D concentrations.

A fitted line from an unadjusted cosinor model is overlaid on the baseline measured values. The mean (annual) 25(OH)D concentration obtained from the model was 38.4 nmol/L (95% CI:36.8–39.9), and the peak-trough difference in annual mean was 38.7 nmol/L (95% CI: 34.2–43.2). A trough occurred between February and March while a peak was between May and June. The amplitude was 19.3 nmol/L, which is equivalent to 49.6% of the annual mean. After adjustment for patients’ gender and age, the predicted mean annual vitamin D concentration was 42.2 nmol/L (95% CI:34.3–49.1).

We used these corrected 25(OH)D concentrations for all further analysis. We compared the concentrations of the analyzed parameters between ESRD and CG and we examined divergence in the analyzed lipid and apolipoprotein parameters in ESRD patients divided according to the clinically significant concentrations of 25(OH)D (those with < 50 nmol/L and ≥ 50 nmol/L) (Table 1).

**Table 1:** Comparison of the lipid parameters between ESRD patients and CG participants and between subgroups based on adjusted 25(OH)D concentrations for seasonal variation (< 50 nmol/L and ≥ 50 nmol/L) in ESRD patients ^ba^significant difference between ESRD and CG; ^b^significant difference between ESRD subgroups. Data are presented as median and interquartile range (IQR); Mann–Whitney U test for independant samples; ApoAI, Apolipoprotein AI; ApoB, apolipoprotein B; CI, confidence interval; CG, Control Group; ESRD, end stage renal disease; HDL-C, high-density lipoprotein; LDL-C, low-density lipoprotein; N, number of subjects; TG, tryglicerides; TC, total cholesterol; 25(OH)D, 25-hydroxy vitamin D

Lipid parameter	CGN = 50	ESRD patients		p
		< 50 nmol/L PN = 167	≥ 50 nmol/L N = 47	
25(OH)D, nmol/L	73.5 (67.0–81.8)	35.1 (28.9–41.9)	52.9 (48.9–59.9)	**< 0.001** ^a^, ^b^
TC, mmol/L	4.32 (4.12–4.65)	5.98 (5.03–6.30)	6.22 (5.98–6.55)	**0.003** ^a^, **0.005** ^b^
HDL-C, mmol/L	1.53 (1.38–1.67)	0.82 (0.68–0.98)	0.88 (0.73–0.98)	**0.039** ^a^, 0.429^b^
LDL-C, mmol/L	2.31 (2.18–2.46)	5.01 (4.63–5.42)	4.99 (4.65–5.14)	**< 0.001** ^a^, 0.142^b^
TG, mmol/L	1.09 (0.91–1.39)	2.13 (1.95–2.78)	2.14 (1.962.54)	**0.003** ^a^, 0.808^b^
ApoB, g/L	0.93 (0.75–1.12)	1.47 (1.31–1.67)	1.49 (1.43–1.67)	0.072^a^, 0.309^b^
ApoAI, g/L	1.89 (1.47–2.09)	1.71 (1.43–1.92)	1.80 (1.63–1.99)	**0.046** ^a^, **0.049** ^b^
Lp(a), g/L	0.21 (0.17–0.24)	0.37 (0.29–0.45)	0.34 (0.30–0.42)	**0.006** ^a^, 0.820^b^
ApoB/ApoAI	0.53 (0.39–0.65)	0.87 (0.73–1.11)	0.87 (0.75–0.95)	**0.011** ^a^, 0.419^b^
LDL–C/HDL–C	1.53 (1.36–1.71)	6.11 (5.14–7.54)	5.55 (4.94–6.57)	**< 0.001** ^a^, 0.256

We found significantly higher concentrations of TC (P = 0.003), LDL-C (P < 0.001), TG (P = 0.003), Lp(a) (P = 0.006), ApoB/ApoAI (P = 0.011) and LDL-C/HDL-C ratio (P < 0.001), and significantly lower concentrations of HDL-C (P = 0.039) and ApoAI (P = 0.046) in ESRD patients compared to CG (Table 1). When we have compared lipid parameters concentrations between ESRD subgroups, we have found significantly higher TC (P = 0.005) and ApoAI (P = 0.049) in ESRD with 25(OH)D concentrations ≥ 50 nmol/L (Table 1).

**Figure 2:**
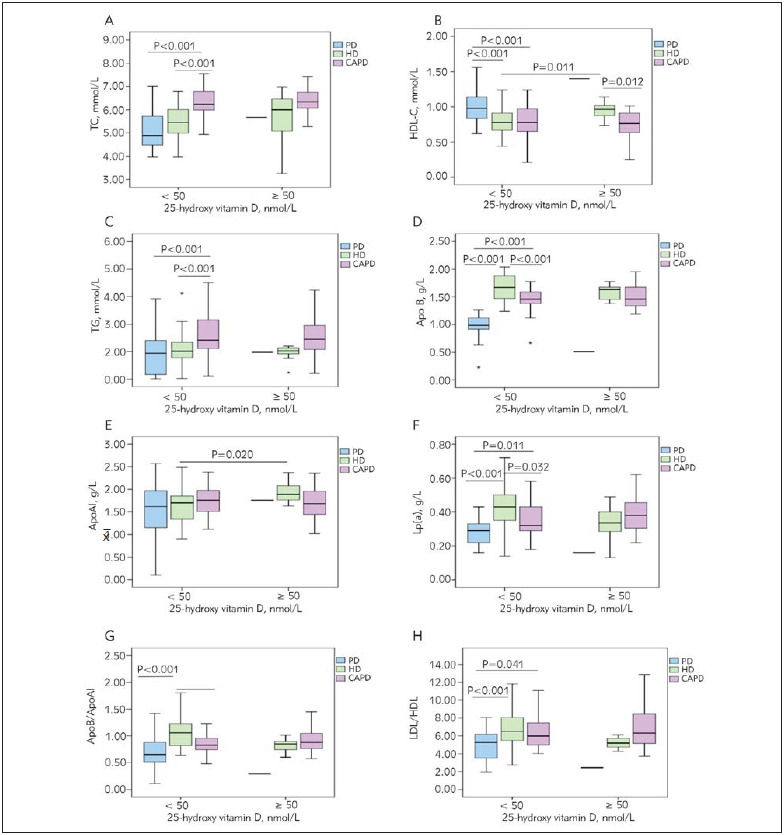
Impact of the maintenance dialysis and dialysis mode in relation to 25-hydroxy vitamin D concentrations on lipid parameters A: TC, total cholesterol; B: HDL-C, high density lipoprotein; C: TG, tryglicerides; D: ApoB, apolipoprotein B; E: ApoAI, apolipoprotein AI; F: Lp(a), lipoprotein (a); G: ApoB/ApoAI ratio and H: LDL-C/HDL-C ratio; PD, predialysis patients; HD, hemodialysis patients; CAPD, patients on continuous ambulatory peritoneal dialysis

In this study, we considered possible effect of dialysis mode on the analysed lipid parameters in relation to corrected 25(OH)D concentrations (< 50 nmol/L and ≥ 50 nmol/L) (Figure 2).

After adjustment of 25(OH)D concentrations for seasonal variation we found that only one PD patient had 25(OH)D concentration above used cut off in this study (55.1 nmol/L). Median and IQR for 25(OH)D concentrations < 50 nmol/L in PD patients was 38.9 nmol/L (33.1-42.1). Median and IQR for 25(OH)D concentrations < 50 nmol/L and ≥ 50 nmol/L in HD and CAPD patients were: 34.1 nmol/L (28.9-39.1), 53.1 nmol/L (48.8 -55.7), 33.9 nmol/L (26.7-41.9) and 52.9 nmol/L (48.9-60.9), respectively.

When we compared lipid parameters in HD patients according to their 25(OH)D status, we found significantly higher HDL-C (0.97 vs. 0.78 mmol/L, P = 0.011) and ApoAI (1.91 vs. 1.70 g/L, P = 0.020) in 25(OH)D ≥ 50 nmol/L HD group. The other analyzed lipid parameters did not differ significantly re garding the 25(OH)D concentrations in the studied groups of patients. In groups with adjusted 25(OH) concentrations ≥ 50 nmol/L, we found significantly higher HDL-C (0.97 vs. 0.77 mmol/L, P = 0.012) in HD patients compared to CAPD group (Figure 2).

However, we have found significant impact of dialysis mode in groups with 25(OH)D concentrations < 50 nmol/L (Figure 2). We have found significantly lower ApoB (0.99 vs. 1.67 g/L, P < 0.001), Lp(a) (0.29 vs. 0.43 g/L, P < 0.001), ApoB/ApoAI (0.65 vs. 1.06, P < 0.001) and LDL-C/HDL-C (5.27 vs. 6.52, P < 0.001), and significantly higher HDL-C (0.98 vs. 0.78, P < 0.001) in PD patients compared to HD group. In addition, PD patients have had significantly lower TC (4.89 vs. 6.23 mmol/L, P < 0.001), TG (1.94 vs. 2.42 mmol/L, P < 0.001), ApoB (0.99 vs. 1.46 g/L, P < 0.001), Lp(a) (0.29 vs. 0.32 g/L, P = 0.11) and LDL-C/HDL-C (5.27 vs. 5.98, P = 0.041), and significantly higher HDL-C (0.98 vs. 0.78 mmol/L, P < 0.001) in comparison with CAPD group, respectively. When we compared HD and CAPD groups with 25(OH)D D concentrations < 50 nmol/L, we have found significantly lower TC (5.46 vs. 6.23 mmol/L, P < 0.001), TG (2.02 vs. 2.42 mmol/L, P < 0.001), and significantly higher ApoB (1.67 vs. 1.46 g/L, P < 0.001), Lp(a) (0.43 vs. 0.32 g/L, P = 0.032) and ApoB/ApoAI (1.06 vs. 0.83, P < 0.001). There was no difference in lipid parameters concentrations in relation to 25(OH)D status in CAPD patients.

## Discussion

The main finding of our study is that there is a significant relationship between serum 25(OH)D concentrations and lipid status and that adequate 25(OH)D concentrations contribute to favorable regulation of lipid status parameters in ESRD patients. While literature data are still controversial about the protective role of 25(OH)D concentrations against CVD, there is an unequivocally confirmed relation between lipid parameters and 25(OH)D concentrations in ESRD patients [19] [20] [21]. However, there is no definitive conclusion on the effect of vitamin D supplementation on lipid concentrations. 25(OH)D concentrations vary seasonally due to highly variable exposure to sunlight, which is why single measurements usually poorly reflect long-term 25(OH)D status. Circulating 25(OH)D concentrations differ substantially over the calendar year, within individuals and general population. Normally the concentrations are lowest at the end of winter and highest at the end of summer. This is likely due to seasonal differences in ultraviolet light exposure [21]. Nevertheless, our study is the first to investigate relationship between adjusted 25(OH)D and lipid status for PD and dialysis patients, with respect to dialysis mode.

When we analyzed overall ESRD patients relative to the CG, resulting lipid concentrations were in agreement with KDIGO recommendations, except TC concentrations wich were significantly higher in ESRD patients compared to the CG. In support of our findings, several previous studies showed that high concentrations of TC might play an important role in the pathogenesis and progression of kidney disease [1] [2] [3]. Significantly higher TC concentrations in 25(OH)D ≥ 50 nmol/L, could support the fact that there was a tendency towards an increase in TC concentrations with supplementation of vitamin D and that vitamin D may have potentially unfavorable effects on lipoprotein metabolism [19]. Opposite to this finding was the Wienstock-Guttman et al. [7] study that discusses sufficient vitamin D levels and its impact on correction of high TC in patients with multiple sclerosis. The question is whether there is a different vitamin D-lipid interdependence concerning different pathophisiology of patients. In this study, prior to measuring 25(OH)D concentrations, we carried out deproteinisation as a standard preanalitical phase of HPLC procedure, so we principally excluded any effect of DBP on 25(OH)D concentrations. However, it still remains possible that DBP affects lipid concentration in ESRD patients. This remains for future studies to reveal. Based on currently available literature, the increase in 25(OH)D concentration from insufficient to optimal range and its effects on lipid status have been varied and inconclusive [4] [10] [13] [22]. Unchanged Lp(a) concentration in different 25(OH)D groups could implicate that this lipoprotein is not clinically significant in prediction of CVD progression and complications in ESRD patients, except if considered with adjustment for dialysis mode. We did not found significant difference in LDL-C concentrations regarding the 25(OH)D concentrations. However, we cannot exclude the formation of qualitatively different molecules from normal LDL-C (small-dense LDL-C, oxidized LDL-C). Previous data indicate that there is a »grey zone« for the Friedewald equation for TG concentrations around 4.45 mmoL/L, that tends to underestimate LDL-C in the setting of high TG concentrations [23] [24]. This is especially critical at low LDL-C concentrations, which could result in undertreatment of high-risk patients. In this study we had only 6% of CAPD patients (total 6 of 92 patients) that had TG concentration above 4.45 mmol/L, but did not have low LDL-C (concentrations from 4.22 to 5.78 mmol/L). There are controversial facts about clinical significance of lipid ratios for prediction of CVD, but generally overall ESRD patients were not studied. However, some studies reported that ApoB/ApoAI ratio is better than LDL-C, and that ApoB/ApoAI increase indicates an increased risk of CVD and may provide some useful information in the differential diagnosis [25] [26]. Our study results support these findings because even though we did not found any difference in ApoB concentrations in ESRD patients regarding 25(OH)D concentrations, nor between ESRD and CG patients, our data showed that ApoB/ApoAI and LDL-C/HDL-C ratio were significantly higher in ESRD patients compared to CG, regardless of 25(OH)D concentrations. Lipid ratios could be an independent cardiovascular risk indicators in ESRD patients, but future studies need to evaluate the potential advantage of integrating them into routine clinical practice.

In addition, we analyzed lipid parameters in relation to 25(OH)D concentrations (< 50 nmol/L and ≥ 50 nmol/L) separately for PD, HD and CAPD patients. Main restriction of this study was that after adjustment of 25(OH) concentrations for seasonal variation, we found only one PD patient that had 25(OH)D concentration ≥ 50 nmol/L. So we could not present reliable relationship between lipids and 25(OH)D concentrations ≥ 50 nmol/L in this particular group. However, lower TC, TG, ApoB, Lp(a) and lipid ratios in PD patients with 25(OH)D < 50 nmol/L regarding the dialysis mode confirm undesirable effect of dialysis on lipid status. Ko et al. reported that lower 25(OH)D concentrations can influence the cardiovascular as well as all-cause mortality in PD patients and that 25(OH)D level could be an equal predictor of patient outcomes in PD and HD patients [27]. Contrary to our findings of significantly higher ApoB concentrations and ApoB/ApoAI ratio in the analysed HD patients with ≥ 50 nmol/L 25(OH)D concentrations compared to 25(OH)D < 50 nmol/L HD patients, some previous studies have presented positive effect of vitamin D supplementation on the lipid parameters of regular hemodialysis patients [28] [29] [30]. Our study results could indicate the additional factors that may affect lipoprotein metabolism in dialysis patients such as treatment with erythropoietin, L-carnitine supplementation, use of low molecular weight heparin and high-flux dialysis. There is evidence that CAPD patients exhibit a more atherogenic concentrations of lipid parameters than HD patients, but we found no significant impact of 25(OH)D on the lipid status of the analysed CAPD patients [31]. Unexpected result in this study was higher HDL-C in HD patients in comparison with CAPD vitamin D sufficient group, that may indicate the HDL-C dys function in CAPD patients. This is in line with the results of Moradi et al. [32] who pointed out that this can further exacerbate the cardiovascular disease burden.

Observation of our study of the inconsiderable impact of the 25(OH)D concentrations on other lipid status parameters in CAPD patients could point to unevenly peritoneal loss of both lipids and vitamin D. However, other facts should be taken into account such as the duration of peritoneal dialysis procedure, vitamin D analogs concentration, and patient's age. Despite the recent explaination that CAPD patients have better quality of life, this patients may present a further increase of CVD risk if not adequately monitored [32] [33] [34]. After using cosinor analysis for seasonal correction of 25(OH)D concentrations, we found a trend of normalization 25(OH)D values, which also refflected its relationship with lipids. More data is needed to improve clinical utility of seasonally adjusted vitamin D level regarding lipid status in ESRD patients.

This study has some limitations. Design of this study was cross-sectional and we were not able to analyze the long-term effects of vitamin D supplementation on lipid parameters in the studied population, although we realize that an interventional study could better explain the causal relationship between variables. Main omission of this study is that we did not measure DBP and evaluate its possible effect on vitamin D and lipid status in studied ESRD patients. In addition, we did not evaluate any inflammatory markers, although these markers have physiological relationships with lipid abnormalities. Potential confounding of therapy with cholesterollowering agents was not examined and we had small number of patients who were on therapy including vitamin D suplementation.

## Conclusion

One important clinical consideration and conclusion of our study is that lipids and lipoproteins as well as lipid ratio parameters is preferably to controlled in relation with 25(OH)D concentrations and separately for PD and dialysis patients. In addition, there is noticeable effect of dialysis mode on lipid parameters. This study findings could be useful for further vitamin D suplementation in ESRD patients in relation to lipid menagement, but further research is necessary to explain whether joint evaluation of 25(OH)D status and lipid abnormalities could improve cardiovascular outcome in ESRD patients.

## List of abbreviations

ApoAI, apolipoprotein AI; ApoB, apolipoprotein B; CVD, cardiovascular disease; CAPD, patients on continuous ambulatory peritoneal dialysis; CG, control group; DBP, vitamin D binding protein; ESRD, end stage renal disease; GFR, glomerular filtration rate; HD, hemodialysis patients; HDL-C, high-density lipoprotein cholesterol; KDIGO, Kidney Disease Improving Global Outcomes; Lp(a), lipoprotein(a); LDL-C, lowdensity lipoprotein cholesterol; PD, predialysis patients; TC, total cholesterol; TG, triglycerides; 25(OH)D, 25-hydroxy vitamin D.
